# Enhancement of Image Quality in Low-Field Knee MR Imaging Using Deep Learning

**DOI:** 10.7759/cureus.71277

**Published:** 2024-10-11

**Authors:** Tsutomu Inaoka, Akihiko Wada, Masayuki Sugeta, Masaru Sonoda, Hiroyuki Nakazawa, Ryosuke Sakai, Hisanori Tomobe, Koichi Nakagawa, Shigeki Aoki, Hitoshi Terada

**Affiliations:** 1 Department of Radiology, Toho University Sakura Medical Center, Sakura, JPN; 2 Department of Radiology, Juntendo University School of Medicine, Tokyo, JPN; 3 Department of Radiology, Seirei Sakura Citizen Hospital, Sakura, JPN; 4 Department of Orthopaedic Surgery, Toho University Sakura Medical Center, Sakura, JPN

**Keywords:** deep learning (dl), diagnostic accuracy, image quality enhancement, knee, low-field strength, mri, musculoskeletal, red-net, super-resolution, u-net

## Abstract

Purpose: The purpose of this study is to investigate the potential of deep learning (DL) techniques to enhance the image quality of low-field knee MR images, with the ultimate goal of approximating the standards of high-field knee MR imaging.

Methods: We analyzed knee MR images collected from 45 patients with knee disorders and six normal subjects using a 3T MR scanner and those collected from 25 patients with knee disorders using a 0.4T MR scanner. Two DL models were developed: a fat-suppression contrast-generation model and a super-resolution model. These DL models were trained using 3T knee MR imaging data and applied to 0.4T knee MR imaging data. Visual assessments of anatomical structures and image noise and abnormality detection with diagnostic confidence levels on the original 0.4T MR images and those after DL enhancement were conducted by two board-certified radiologists. Statistical analyses were performed using McNemar’s test and the Wilcoxon signed-rank test.

Results: DL-enhanced MR images significantly improved the depiction of anatomical structures and reduced image noise compared to the original MR images. The number of abnormal findings detected and the diagnostic confidence levels were higher in the DL-enhanced MR images, indicating the potential for more accurate diagnoses.

Conclusion: DL techniques effectively enhance the image quality of low-field knee MR images by leveraging 3T MR imaging data. This enhancement significantly improves image quality and diagnostic confidence levels, making low-field MR images much more reliable for detecting abnormalities. This advancement offers a useful alternative for clinical settings, especially in resource-limited environments, without compromising diagnostic accuracy.

## Introduction

Currently, magnetic resonance imaging (MRI) undeniably plays a crucial role in the diagnosis of the musculoskeletal system. As a non-invasive diagnostic imaging tool, MRI excels in providing detailed evaluations, particularly in joint assessments [1,2]. T2-weighted images (T2WI) and proton-density-weighted images (PDWI) are crucial for arcuate MRI-based diagnosis of the joints. Additionally, fat-suppression techniques are essential as they eliminate signals from fat tissue and bone marrow on T2WI and PDWI, enhancing the detection accuracy of abnormalities in the articular cartilage, meniscus, ligaments, bone marrow, and soft tissues [3-7]. Therefore, these capabilities facilitate the formulation of accurate diagnoses and treatment plans based on the observed findings.

Low-field MRI is considered a cost-effective and clinically user-friendly diagnostic imaging tool and is highly regarded in medical practice [8,9]. Its usefulness has extended even to the musculoskeletal system [10-12]. However, low-field MRI has its drawbacks, most notably a reduction in image quality and contrast compared to high-field MRI. This limitation is particularly evident in fat-suppression magnetic resonance (MR) images [13-15]. In low-field MRI, distinguishing signals from fat and water protons becomes challenging. Although there is a desire to use frequency-selective techniques in fat-suppression imaging to maintain image quality and contrast of T2WI and PDWI, the common use of non-frequency-selective fat-suppression techniques, such as short-tau inversion recovery (STIR), remains prevalent [7,13,14]. However, STIR has limitations in diagnosing internal derangement in joints due to its lower quality and resolution.

In recent years, there has been increasing interest in utilizing deep learning (DL) to improve image quality and contrast, as well as to shorten examination times, in medical practice. Indeed, DL methods have shown promising results in clinical applications, delivering excellent outcomes in both image quality and contrast, particularly in knee MRI [16-24]. Additional advancements have been made in the image-generation techniques themselves [25]. For example, we have successfully experimented with generating fat-suppression images of the knee from the original 3T MRI data and have reported the feasibility of this technique [26]. Recently, several studies have applied DL-based image quality and contrast enhancement techniques to images acquired with low-field MRI [27-29]. In the present study, we aim to apply these techniques to enhance the quality and contrast of fat-suppression images obtained from low-field MRI, seeking to approximate the standards achieved with high-field MRI.

## Materials and methods

This study was conducted with the approval of the Ethics Committee of the Toho University Sakura Medical Center (S23040_S20064_S20015). The use of clinical data for this research was disclosed on the institutional website, and the potential participants were given the opportunity to decline to further enroll in this study. In managing data from external entities, strict adherence was maintained to the standards set forth by the respective external facilities as well as our institute.

Sample selection

For MRI data at high-field strength, a total of 45 knee MRI studies in 45 patients with knee disorders (mean age 54.6 ± 20.3 years; 16 males/29 females; 21 right/24 left) performed with a 3T MR scanner (Magnetom Skyra, Siemens Healthcare, Erlangen, Germany) with an eight-channel knee coil between April 2020 and July 2020 were included. Cases after ligament reconstruction were excluded. Additionally, 12 knee MRI studies in six normal subjects without any knee symptoms or history of disorders (mean age 34.2 ± 9.5 years; four males/two females; 6 right/6 left) conducted using the same 3T MR scanner with an eight-channel knee coil in March 2020 were included. Written informed consent was obtained from all the normal subjects.

For MRI data at low-field strength, a total of 25 knee MRI studies in 25 patients suspected of having knee disorders obtained with a 0.4T MR scanner (APERTO Lucent, Fujifilm Medical Systems, Japan) in May 2021 were included. The patient information, including age, gender, and clinical diagnoses, could not be disclosed due to the regulations of the participating external facilities. This information was strictly managed by one of the authors (M.S.).

The imaging parameters at 3T and 0.4T are shown in Table 1.

**Table 1:** Imaging parameters of knee MRI at 3 Tesla and 0.4 Tesla T1WI: T1-weighted image; T2WI: T2-weighted image; FS: fat-suppressed; STIR: short-tau inversion recovery; TR: repetition time; TE: echo time; TI: inversion recovery time; FOV: field of view.

	3 Tesla			0.4 Tesla		
	T1WI	T2WI	FS-T2WI	T1WI	T2WI	STIR
Plane	Sagittal	Sagittal	Sagittal	Sagittal	Sagittal	Sagittal
TR/TE (ms)	520/10	4500/31	4500/31	380/19	4792/90	6013/20
TI (ms)						110
FOV (mm)	160	160	160	160	160	160
Slice thickness (mm)	3	3	3	3	3	3
Interval (mm)	3.3	3.3	3.3	3.5	3.5	3.5
Matrix	448×291	448×291	448×291	320×224	288×192	256×192

All the images were extracted in Digital Imaging and Communications in Medicine (DICOM) file format and converted to 8-bit greyscale Portable Network Graphics (PNG) format. The image resolution was set to 448×448 pixels for 3T MR images and to 256×256 pixels for 0.4T MR images.

DL model

The DL model used two-dimensional convolutional neural networks on the open-source Neural Network Console ver.2.1 deep learning library, which was commercially developed (Sony Network Communications, Tokyo: https://dl.sony.com) based on the Python programming language (version 3.6.3; Python Software Foundation, Wilmington, DE) and run on a computer (eX. computer, Windows 10 operating system) with an AMD Ryzen 9 3950X 3.5 GHz processor, 64 GB RAM, and an NVIDIA GeForce RTX 2080Ti 11 GB graphics processing unit (NVIDIA, Santa Clara CA).

**Figure 1:**
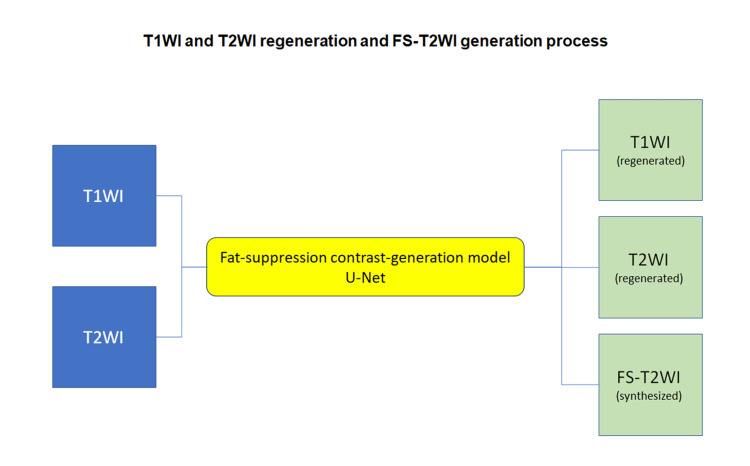
Fat-suppression contrast-generation model The diagram illustrates the structure of a deep learning model designed for generating fat-suppressed T2-weighted images (FS-T2WI) from original T1-weighted images (T1WI) and T2-weighted images (T2WI), as well as for restoring T1WI and T2WI. The model architecture incorporates a U-Net (yellow box), which plays a crucial role in the image generation process.

We have developed two DL models: one is a fat-suppression contrast-generation model, and the other is a super-resolution model. The fat-suppression contrast-generation model is designed to synthesize fat-suppressed T2-weighted images (FS-T2WI) from original T1-weighted images (T1WI) and T2WI, as well as to restore T1WI and T2WI, utilizing a U-Net model (Figure 1).

**Figure 2:**
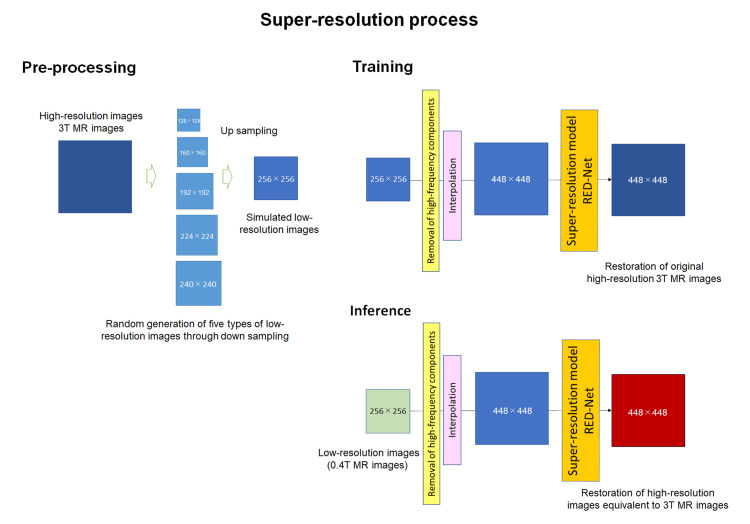
Super-resolution model The diagram illustrates the structure of a super-resolution model, which is composed of three parts as follows: Pre-processing: High-resolution images from 3T MR images are randomly downsampled and then upsampled to generate low-resolution images that mimic low-field MR images. As a result, 256×256 matrix images are created. Training: The low-resolution images generated from pre-processing are restored to high-resolution images from 3T MR images. In this process, high-frequency components are first removed (yellow box), followed by interpolation processing (pink box). The images created through this process are then subjected to super-resolution processing using RED-Net (orange box), which is a crucial step. Inference: Using this process, low-field MR images (low-resolution images) are input and processed to achieve high-resolution images. As a result, 448×448 matrix images are created.

On the other hand, the super-resolution model aims to restore the original high-resolution images from downsampled low-resolution images and enhance the image quality and contrast. Then, the encoder-decoder model of RED-Net for the super-resolution process was used (Figure 2).

The super-resolution model was applied to T1WI, T2WI, and FS-T2WI generated from 0.4T MRI data. Thus, the T1WI, T2WI, and FS-T2WI generated by the fat-suppression contrast-generation model from the original T1WI and T2WI were processed by the super-resolution model to enhance the image quality and contrast. We utilized knee MRI data acquired with a 3T MR scanner for the training of these DL models. To enhance the learning efficiency of the super-resolution model, the high-frequency components of the images were removed and normalized. Additionally, we optimized the learning process for generating high-frequency components to match the lower-resolution images created from downsampled 3T MR images to 0.4T MR images. This process was also adopted for lower-resolution images from 0.4T MRI. Therefore, these processes were employed for knee MR images acquired at low-field strengths to resemble knee MR images acquired at high-field strengths.

Image assessment

Knee MR images generated after enhancing image quality and contrast were compared to the original knee MR images obtained with a 0.4T MR scanner. Initially, a visual assessment was conducted by two board-certified radiologists (M.S., T.I.) to evaluate the image quality and contrast of the original T1WI, T2WI, and STIR at 0.4T, as well as the T1WI, T2WI and FS-T2WI generated through the DL processes. The visual assessment was performed on a 31.5-inch liquid crystal display monitor (screen ratio 16:9, resolution 2560×1440, 3.7 megapixels) (IIyama, Japan). The image resolution of both the original and generated knee MR images was standardized to 448×448 pixels.

As part of the assessment criteria for image quality and contrast, we evaluated the depiction of anatomical structures and image noise. Specifically, the sharpness of contours and internal structures was assessed for the anatomical depiction. Subjective signal-to-noise ratio and roughness of the images were used to evaluate image noise. The image quality assessment utilized a five-point scale, with 1 corresponding to ‘very poor’, 2 to ‘bad’, 3 to ‘sufficient’, 4 to ‘good’, and 5 to ‘very good’. Furthermore, we evaluated the presence or absence of abnormal finding in the structures, including the articular cartilage (femur, tibia, and patella), meniscus (medial and lateral), cruciate ligament (anterior and posterior), bone marrow (femur, tibia, and patella), muscle (thigh and calf), and joint effusion, using a six-grade scale, with 1 corresponding to ‘definitely normal’, 2 to ‘possibly normal’, 3 to ‘probably normal’, 4 to ‘probably abnormal’, 5 to ‘possibly abnormal’, and 6 to ‘definitely abnormal’. Based on these gradings, the diagnostic confidence levels were determined and categorized into three grades, with 3 corresponding to ‘definite’, 2 to ‘possible', and 1 to ‘probable’.

Statistical analysis

The visualization of anatomical structures and image noise on the original MR images (T1WI, T2WI, and STIR) and those after enhancing image quality and contrast by DL (T1WI, T2WI, and FS-T2WI) were analyzed using McNemar’s test. McNemar’s test was used to analyze the numbers of abnormal findings detected by two readers, as well as the presence or absence of abnormal findings in the structures on the original images and on the images generated after enhancement of image quality and contrast with DL. For the confidence level of diagnosis, the Wilcoxon signed-rank test was used. Statistical analyses were performed using IBM SPSS Statistics for Windows, Version 28 (Released 2023; IBM Corp., Armonk, New York, United States). A p-value less than 0.05 was considered to be statistically significant.

## Results

Visual assessment of image quality

**Figure 3:**
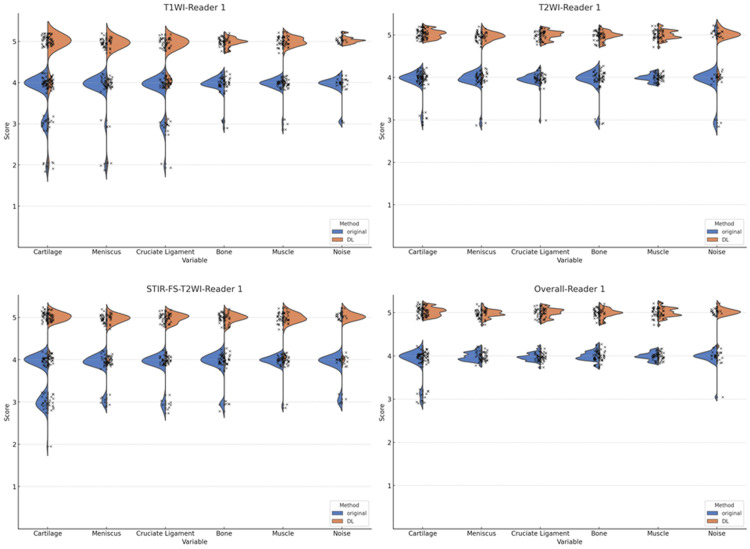
Violin plots of the image quality assessment by reader 1 Statistically significant differences between the original and deep learning (DL)-enhanced images are observed in the image quality for anatomical structures and image noise in T1WI, T2WI, fat-suppressed images (STIR-FS-T2WI), and overall (p<0.001).

**Figure 4:**
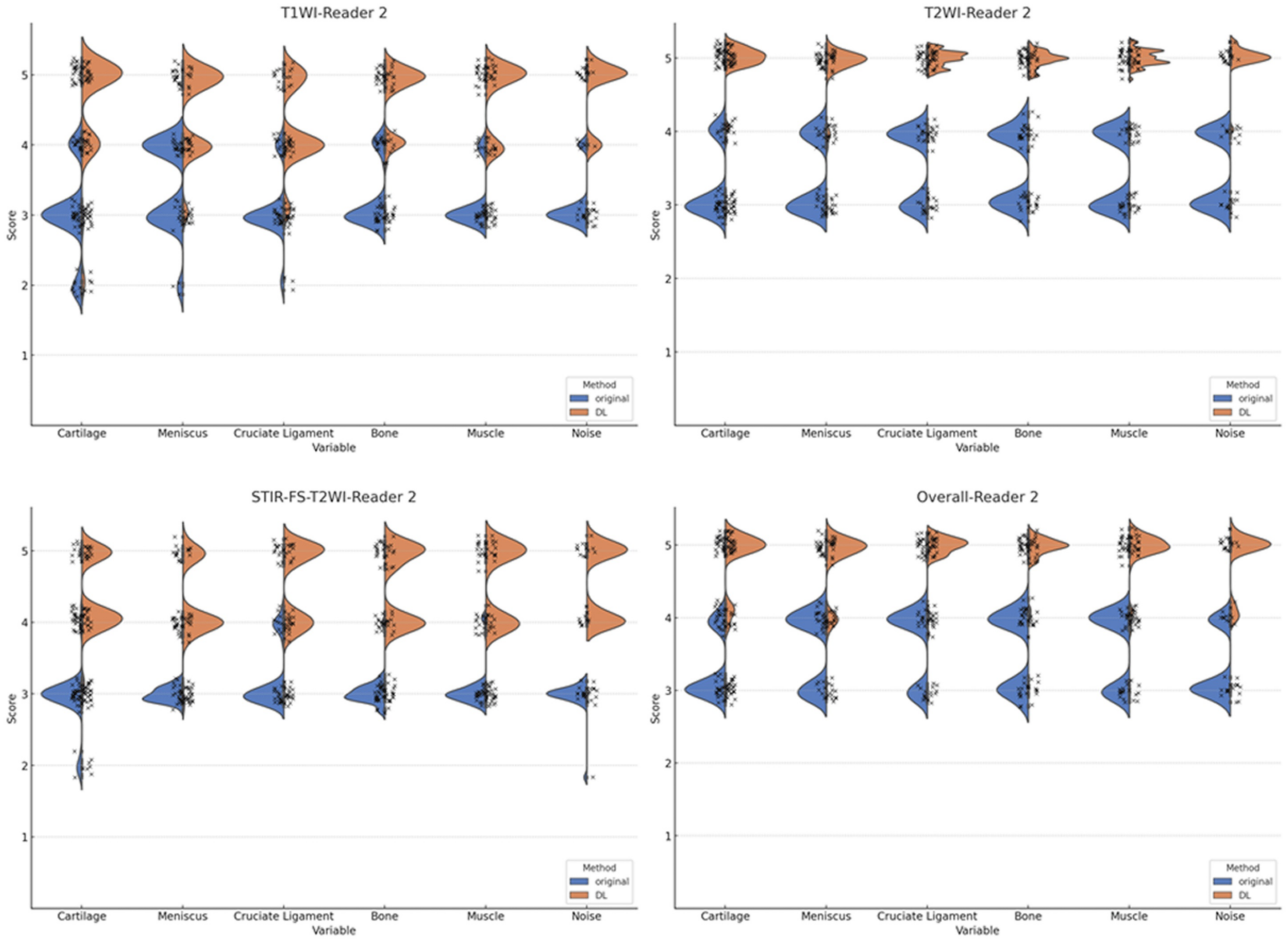
Violin plots of the image quality assessment by reader 2 Statistically significant differences between the original and deep learning (DL)-enhanced images are observed in the image quality for anatomical structures and image noise in T1WI, T2WI, fat-suppressed images (STIR-FS-T2WI), and overall (p<0.001).

The visual assessments of the depiction of anatomical structures and image noise on the original images and those after enhancing image quality and contrast by DL, as evaluated by the two readers, are shown in Figures 3, 4.

Statistically significant differences between the original and DL-enhanced images were observed in the image quality and contrast for the anatomical structures and image noise in T1WI, T2WI, fat-suppressed images, and overall by the two readers. 

Abnormal findings in the structures

Table 2 shows the numbers of abnormal findings in the structures detected on the original and DL-enhanced images by the two readers. Statistically significant differences were found in the category of cartilage for reader 2 (p=0.001) and in the category of muscle and soft tissues for both readers (p<0.05).

**Table 2:** Number of abnormal findings in the structures detected by the two readers Cartilage: articular cartilage in the femur, tibia, and patella; meniscus: medial and lateral meniscus; cruciate ligament: anterior and posterior cruciate ligaments. Variables are expressed as the number of abnormal findings detected. Original: original images, DL: deep learning-enhanced images.

	Method	Number of abnormal findings detected/total number	p-value
Cartilage			
Reader 1	Original	53/75	0.625
	DL	55/75	
Reader 2	Original	50/75	0.001
	DL	64/75	
Meniscus			
Reader 1	Original	17/50	1.000
	DL	18/50	
Reader 2	Original	22/50	0.625
	DL	24/50	
Cruciate ligament			
Reader 1	Original	8/50	1.000
	DL	8/50	
Reader 2	Original	12/50	1.000
	DL	13/50	
Bone			
Reader 1	Original	37/75	1.000
	DL	37/75	
Reader 2	Original	45/75	1.000
	DL	46/75	
Muscle and soft tissues			
Reader 1	Original	15/50	0.041
	DL	21/50	
Reader 2	Original	35/50	0.031
	DL	41/50	

Grade of findings in the structures and confidence level of diagnosis

**Figure 5:**
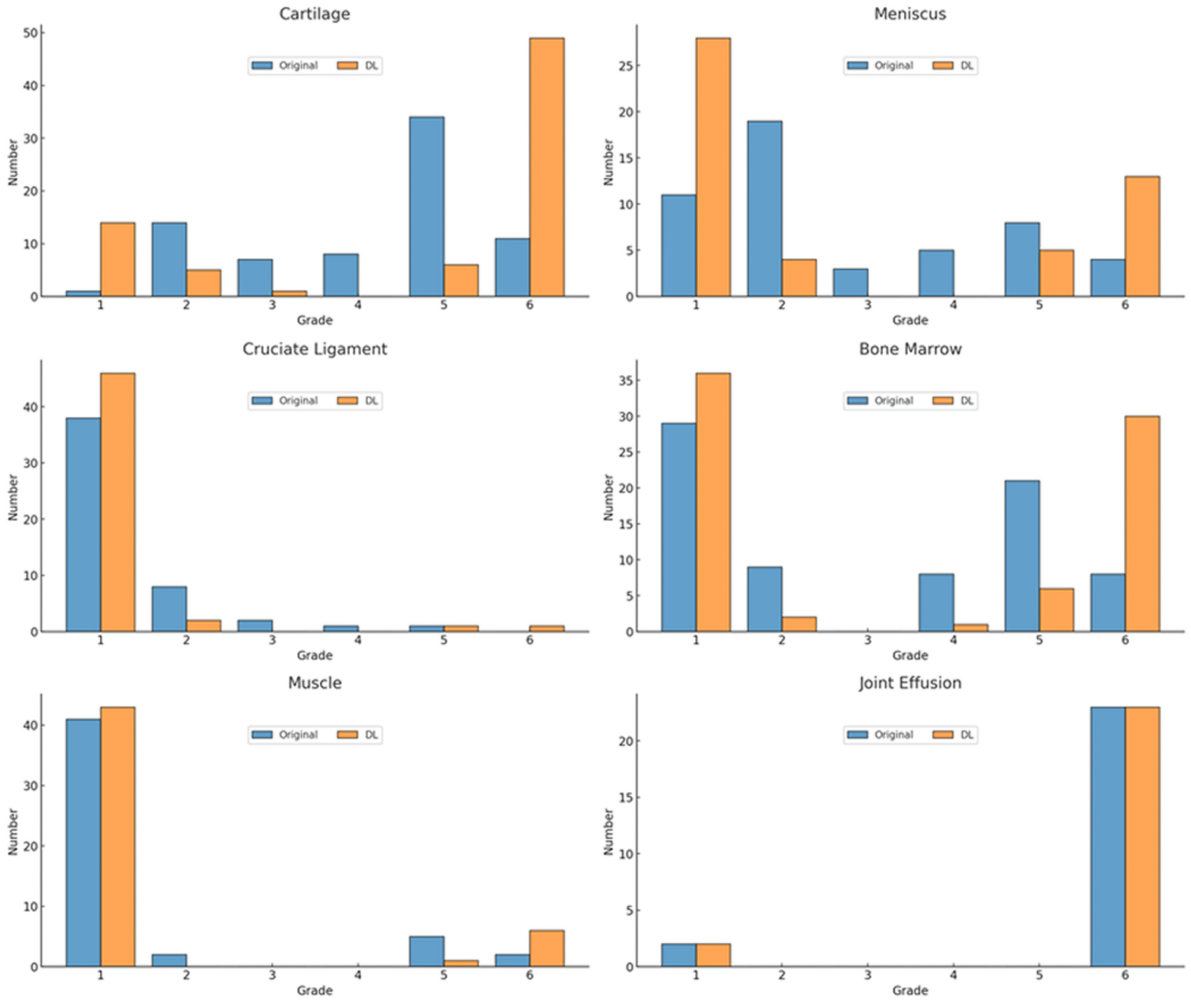
Findings in the structures graded by reader 1 The vertical axis represents the number of abnormal findings and the horizontal axis represents the six-grade scale.

**Figure 6:**
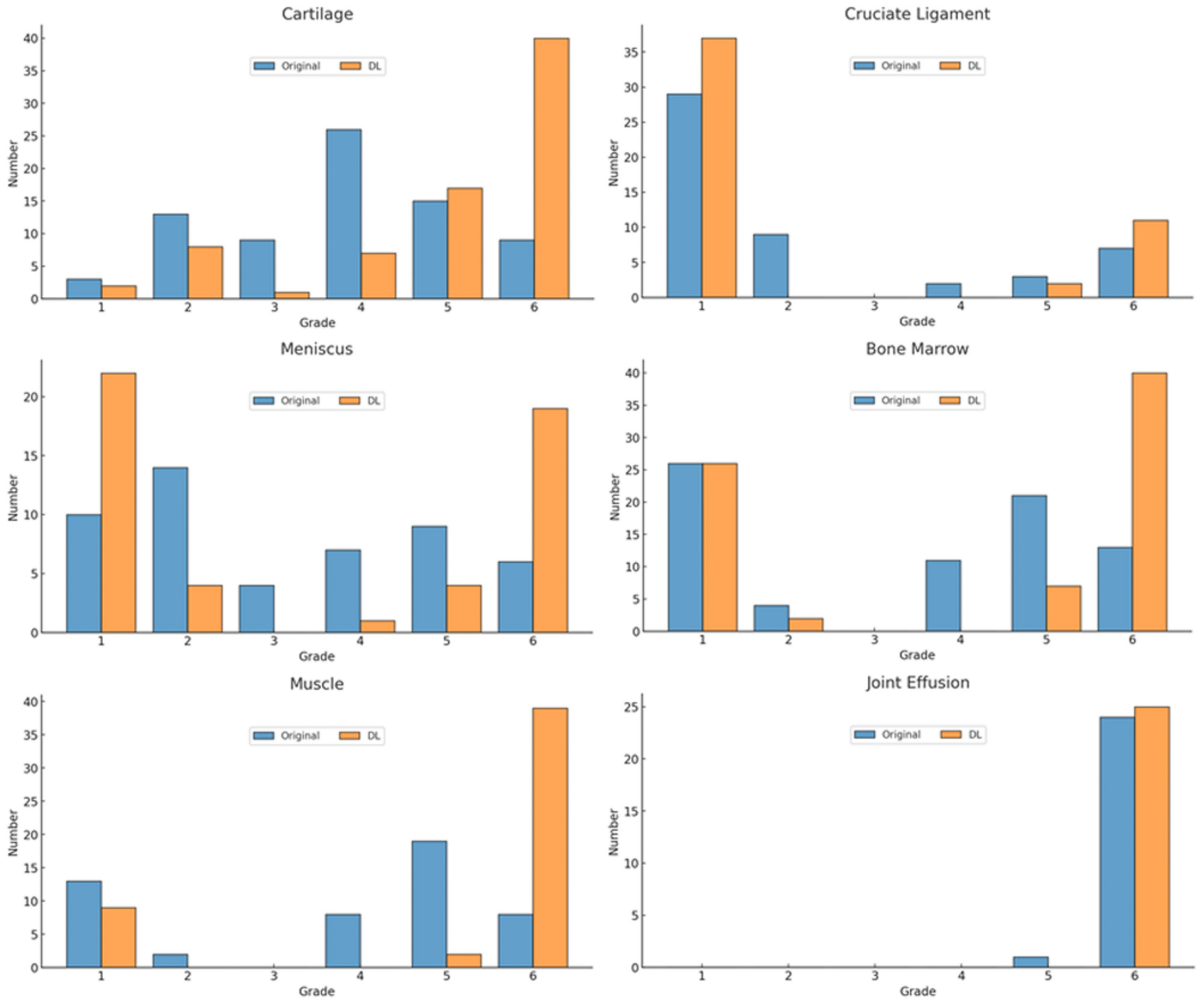
Findings in the structures graded by reader 2 The vertical axis represents the number of abnormal findings and the horizontal axis represents the six-grade scale.

The distributions of normal and abnormal findings in the structures on the original and DL-enhanced images, as scored by the two readers, are shown in Figures 5, 6.

**Figure 7:**
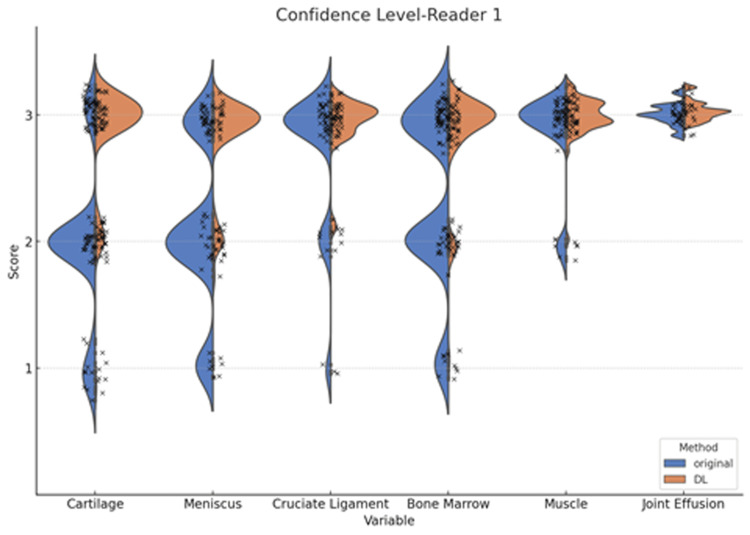
Violin plots of the diagnostic confidence level by reader 1 Statistically significant differences between the original and deep learning (DL)-enhanced images are observed in the diagnosis of the cartilage, meniscus, cruciate ligament, bone marrow, and muscle (p<0.001), but not in the joint effusion.

**Figure 8:**
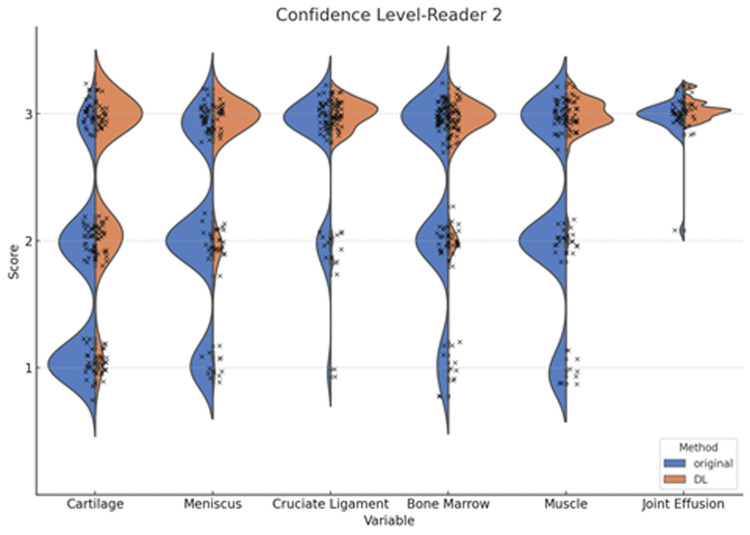
Violin plots of the diagnostic confidence level by reader 2 Statistically significant differences between the original and deep learning (DL)-enhanced images are observed in the diagnosis of the cartilage, meniscus, cruciate ligament, bone marrow, and muscle (p<0.001), but not in the joint effusion.

In addition, the diagnostic confidence levels in normal and abnormal findings detected on the original and DL-enhanced images by the two readers are demonstrated in Figures 7, 8.

Statistically significant differences between the original and DL-enhanced images were observed in the diagnosis of the cartilage, meniscus, cruciate ligament, bone marrow, and muscle (p<0.001), but not in the joint effusion.

**Figure 9:**
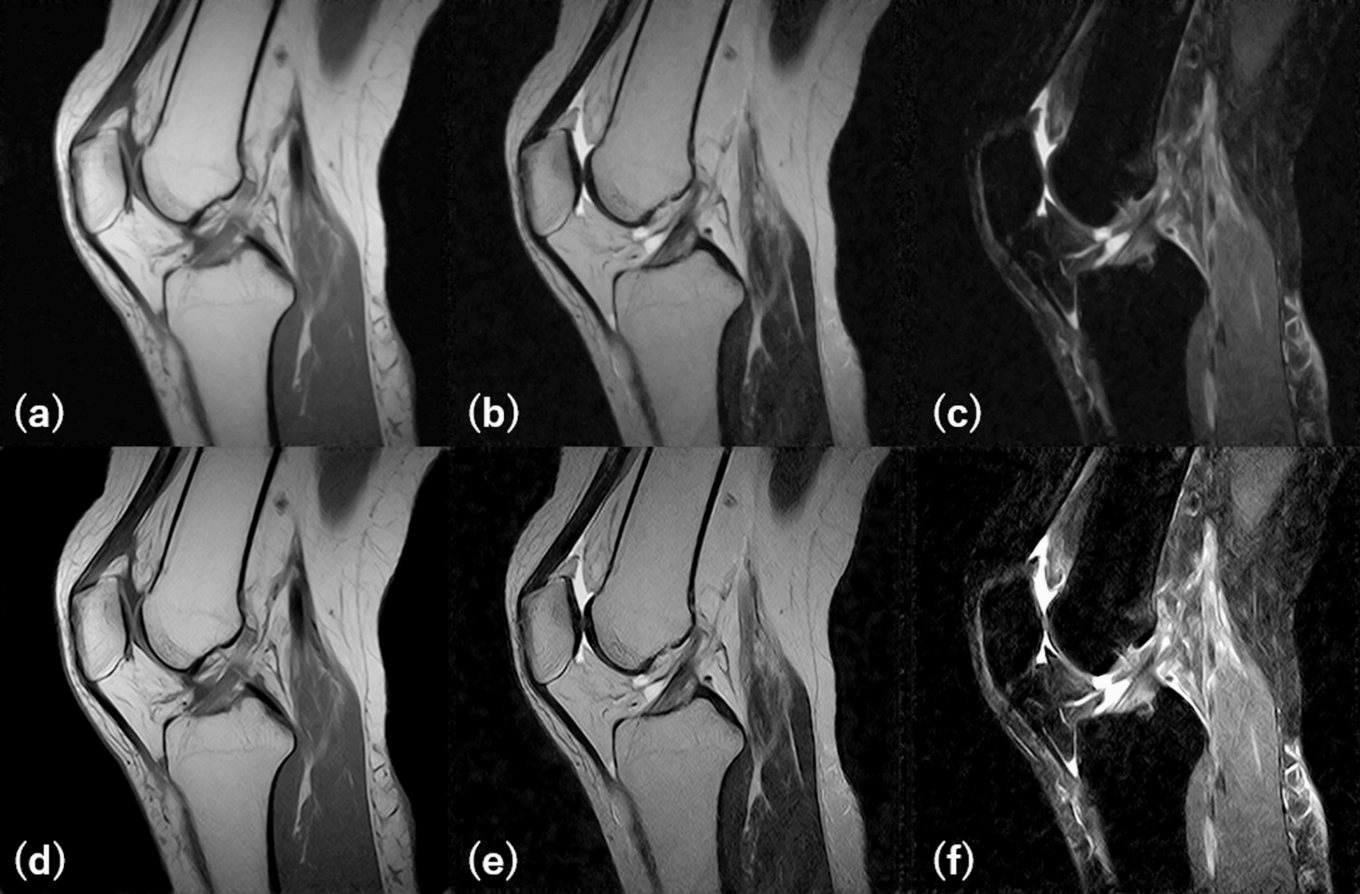
Original and deep learning-enhanced images The top row shows original images, and the bottom row shows deep learning (DL)-enhanced images. (a)T1WI(0.4T), (b)T2WI(0.4T), (c)STIR(0.4T), (d) T1WI (DL-enhanced), (e) T2WI (DL-enhanced), and FS-T2WI (DL-synthesized, DL-enhanced). Image quality and contrast are improved in all the DL-enhanced images compared to the original images. Regarding the overall evaluation of image quality, reader 1 rated the original images as 4 and the DL-enhanced images as 5, and reader 2 rated the original images as 3 and the DL-enhanced images as 5.

**Figure 10:**
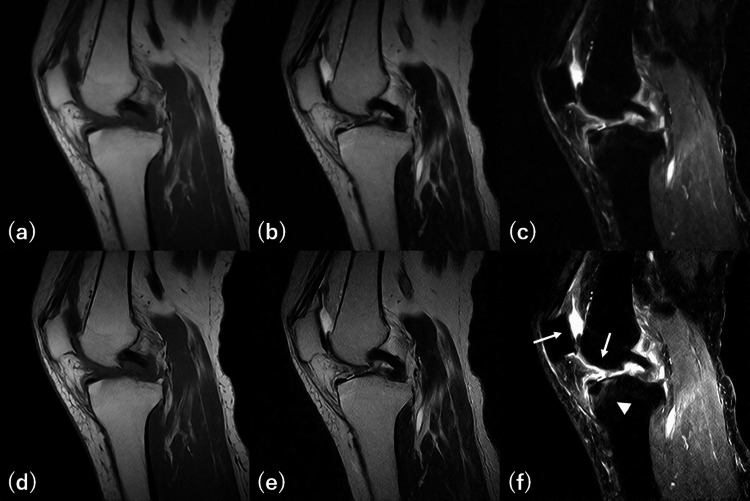
Original and deep learning-enhanced images The top row shows original images, and the bottom row shows deep learning (DL)-enhanced images. (a) T1WI (0.4T), (b) T2WI (0.4T), (c) STIR (0.4T), (d) T1WI (DL-enhanced), (e) T2WI (DL-enhanced), and (f) FS-T2WI (DL-synthesized, DL-enhanced). Image quality and contrast are improved in all the DL-enhanced images. Focusing on the DL-enhanced FS-T2WI (f), thinning and defects of the articular cartilages of the patellofemoral joint are more clearly depicted (white arrows) compared to the original image. Additionally, a bone marrow edema on the anterosuperior aspect of the tibia is also more clearly depicted (arrowhead). Reader 1 rated the articular cartilages of the patellofemoral joint as 6 and the bone marrow of the tibia as 5 on the original images, but rated the articular cartilages of the patellofemoral joint as 6 and the bone marrow of the tibia as 6 on the DL-enhanced images. Reader 2 rated the articular cartilages of the patellofemoral joint as 4 and the bone marrow of the tibia as 5 on the original images, but rated the articular cartilages of the patellofemoral joint as 6 and the bone marrow of the tibia as 6 on the DL-enhanced images.

The representative images are shown in Figures 9, 10.

## Discussion

The findings from our study provide compelling evidence that DL techniques significantly enhance the image quality and diagnostic confidence of low-field MRI, making it a more viable and effective tool for musculoskeletal assessments. The data clearly demonstrate that DL-enhanced images offer superior visualization of anatomical structures and reduced image noise compared to the original low-field MR images. This improvement is critical in clinical settings where high diagnostic accuracy is required for effective patient management.

Figures 3, 4 highlight the substantial improvement in the subjective image quality scores across all categories (cartilage, meniscus, cruciate ligament, bone, and muscle) when using DL-enhanced images compared to the original images. This enhancement is particularly noteworthy in the context of low-field MRI, which typically suffers from reduced image quality and contrast.

Table 2 further shows the benefits of DL-enhanced imaging. The enhancements led to an increase in the number of abnormal findings detected, especially in the cartilage and soft tissue categories. Reader 2, in particular, detected a significantly higher number of abnormal findings in cartilage when using DL-enhanced images (p=0.001). This suggests that DL can enhance the detection sensitivity of low-field MRI, potentially leading to earlier and more accurate diagnoses.

The diagnostic confidence levels showed marked improvement with DL enhancement. Both readers reported higher confidence levels in their diagnoses when using DL-enhanced images across all categories, except for joint effusion. This is crucial for clinical decision-making, as higher confidence levels correlate with more reliable and accurate diagnoses, reducing the likelihood of misdiagnosis and ensuring better patient outcomes.

A key aspect of our study was the enhancement of fat-suppressed images, which are crucial for accurate diagnosis in joint MRI. Low-field MRI often struggles with fat suppression due to its lower field strength, leading to reduced image quality and contrast. Our DL models specifically targeted this issue by generating high-quality fat-suppressed images (FS-T2WI) from original T1WI and T2WI. The results showed significant improvements in the quality of the fat-suppressed images (FS-T2WI). This is particularly beneficial for detecting abnormalities that are otherwise challenging to identify with low-field MRI.

The DL models employed in this study, specifically the fat-suppression contrast-generation model (U-Net) and the super-resolution model (RED-Net) [30], demonstrated significant effectiveness in enhancing low-field MR images. The fat-suppression contrast-generation model effectively synthesized high-quality FS-T2WI from T1WI and T2WI, addressing the limitations of conventional fat-suppression techniques in low-field MRI.

One of the key strengths of our study is the use of the RED-Net model for super-resolution. RED-Net is particularly effective in fields such as image denoising and inpainting [30]. It is a highly useful architecture in DL-based image processing research and application development. RED-Net is effective in restoring high-resolution images from low-resolution counterparts. RED-Net leverages residual connections, which help mitigate the vanishing gradient problem commonly encountered in deep networks. This allows for better gradient flow during training, leading to more stable and faster convergence. The encoder-decoder structure of RED-Net captures both high-level contextual information and fine-grained details, enhancing overall image quality. By utilizing high-frequency component learning, RED-Net effectively restores details often lost in low-resolution images. This capability is crucial for low-field MRI, where the inherent resolution is limited compared to high-field MRI. Integrating RED-Net significantly enhanced the image quality of low-field MR images, resulting in images that were not only visually superior but also diagnostically more reliable. This improvement was evidenced by the increased detection of abnormal findings and higher diagnostic confidence levels.

Despite the promising results, our study has some limitations. The sample size, particularly for the low-field knee MRI group, was relatively small. Additionally, this study was conducted using only sagittal images. Future studies with larger cohorts are necessary to validate our findings and establish the generalizability of DL-enhanced MRI. Furthermore, while our study focused on knee MRI, further research is needed to explore the effectiveness of DL enhancement in other anatomical regions and for different types of musculoskeletal disorders.

Another limitation is that a comparison between images generated using DL from a 0.4T MR scanner and images taken with a 3T MR scanner was not conducted. It may be necessary to compare these images in the same subjects. Our study did not have pathological proof; therefore, future research should incorporate histopathological correlation to confirm the accuracy and reliability of DL-enhanced knee MRI.

In addition, future research should investigate the long-term clinical outcomes of using DL-enhanced low-field MRI in routine practice. This includes assessing the impact on patient management decisions, treatment outcomes, and overall healthcare costs. Moreover, as DL algorithms continue to evolve, ongoing updates and refinements to the models will be necessary to ensure they keep pace with advances in MRI technology and clinical requirements. We believe that, by bridging the gap between low and high-field MRI, DL enhancement paves the way for more accurate, efficient, and cost-effective patient care.

## Conclusions

In conclusion, our study demonstrates that DL techniques, particularly the use of RED-Net for super-resolution, can significantly enhance the image quality and diagnostic confidence of low-field knee MRI, especially in fat-suppressed images. This advancement has the potential to improve the accessibility and effectiveness of musculoskeletal imaging, providing high-quality diagnostic tools to a broader range of clinical settings.
